# Microcins reveal natural mechanisms of bacterial manipulation to inform therapeutic development

**DOI:** 10.1099/mic.0.001175

**Published:** 2022-04-19

**Authors:** Jennifer Kristen Parker, Bryan William Davies

**Affiliations:** ^1^​ Department of Molecular Biosciences, The University of Texas at Austin, Austin, Texas, USA; ^2^​ John Ring LaMontagne Center for Infectious Diseases, The University of Texas at Austin, Austin, Texas, USA

**Keywords:** microcin, bacteriocin, therapeutic, antimicrobial, Gram-negative, membrane

## Abstract

Microcins are an understudied and poorly characterized class of antimicrobial peptides. Despite the existence of only 15 examples, all identified from the *Enterobacteriaceae *, microcins display diversity in sequence, structure, target cell uptake, cytotoxic mechanism of action and target specificity. Collectively, these features describe some of the unique means nature has contrived for molecules to cross the ‘impermeable’ barrier of the Gram-negative bacterial outer membrane and inflict cytotoxic effects. Microcins appear to be widely dispersed among different species and in different environments, where they function in regulating microbial communities in diverse ways, including through competition. Growing evidence suggests that microcins may be adapted for therapeutic uses such as antimicrobial drugs, microbiome modulators or facilitators of peptide uptake into cells. Advancing our biological, ecological and biochemical understanding of the roles of microcins in bacterial interactions, and learning how to regulate and modify microcin activity, is essential to enable such therapeutic applications.

## Introduction

Microbial community dynamics are increasingly appreciated as significant drivers of disease [1, 2]. Models of infection which are reduced to the interactions between a single microbe and its host fail to account for the diverse impacts of the surrounding microbiota in mediating this relationship. Microbial communities perform essential functions to promote and maintain balance in their particular ecosystems. A resilient microbiome may be able to resist invasion by pathogens or suppress overgrowth of pathobionts, while a dysbiotic one may be less capable of doing so [3–5].

As with all ecological communities, interactions such as competition can serve to stabilize the microbiome or skew it towards a dysbiotic state [6, 7]. Understanding the diverse tools that bacteria use to antagonize or otherwise interact with each other will help to elucidate the ecology of disease and lead to tools for disease treatment and prevention. The production and release of antibacterials is one of the primary mechanisms by which bacteria compete with each other [8]. Bacteria secrete a variety of antimicrobial small molecules, proteins and peptides (<100 aa in length [9]). The full range of these naturally secreted products is unknown [10]. Gram-negative bacteria, in particular, are underexplored in terms of their natural diversity of antibiotics [11]. Yet, Gram-negative bacteria are often overrepresented among pathogens of growing concern due to the increasing prevalence of antibiotic resistance. Exploring the native collection of tools that Gram-negative bacteria use for competition could inform the ways in which diseases and microbial community imbalances are treated.

Bacteriocins are a particularly large and diverse class of ribosomally synthesized antibacterial proteins and peptides. They can be post-translationally modified, or not, and have a wide variety of structures and functions. Gram-positive bacteriocins have been studied extensively, though by no means exhaustively, particularly with regardsto potential antibacterial applications [12]. Identified Gram-positive bacteriocins far outnumber Gram-negative bacteriocins, as can be seen in both the BAGEL4 [13] and BACTIBASE [14] bacteriocin databases. Bacteriocins produced by members of the *Firmicutes *, especially the lactic acid bacteria (LAB) of this phylum [15], are abundant in these databases. Numerous LAB bacteriocins, such as the U.S. Food and Drug Administration-approved food preservative, nisin [16], have been examined in detail for therapeutic and other commercial uses [17, 18].

Among Gram-negative bacteria, the majority of bacteriocins have been identified among the *Enterobacteriaceae *. The *Enterobacteriaceae* are a diverse family of Gram-negative bacteria, many of which inhabit the human microbiome as commensals or pathogens. There are two classes of bacteriocins specific to the *Enterobacteriaceae *: the larger colicins (26–75 kDa [19, 20]) and the smaller microcins (1–9 kDa [21, 22]). Microcins have been poorly examined relative to colicins [23]. Only 15 microcins have been identified, and their modes of uptake and mechanisms of antibacterial action are, in general, poorly characterized.

Despite this dearth of information, there is a disproportionate amount of evidence that microcins play a natural role in microbiome modulation and have the potential to be harnessed as therapeutics. This review aims to summarize current evidence supporting the antibacterial functions of microcins, their potential for therapeutic applications, and the information gaps that must be filled to enable this. A more thorough understanding is needed of (1) microcin natural diversity, (2) microcin modes of uptake and mechanisms of action, and (3) the effects of different microcins on individual microbes and the microbial community. This information can provide the missing foundation to allow systematic exploration of microcins for use in microbiome therapies or as leads to new standalone antibacterial treatments.

## Classification

**Fig. 1.**
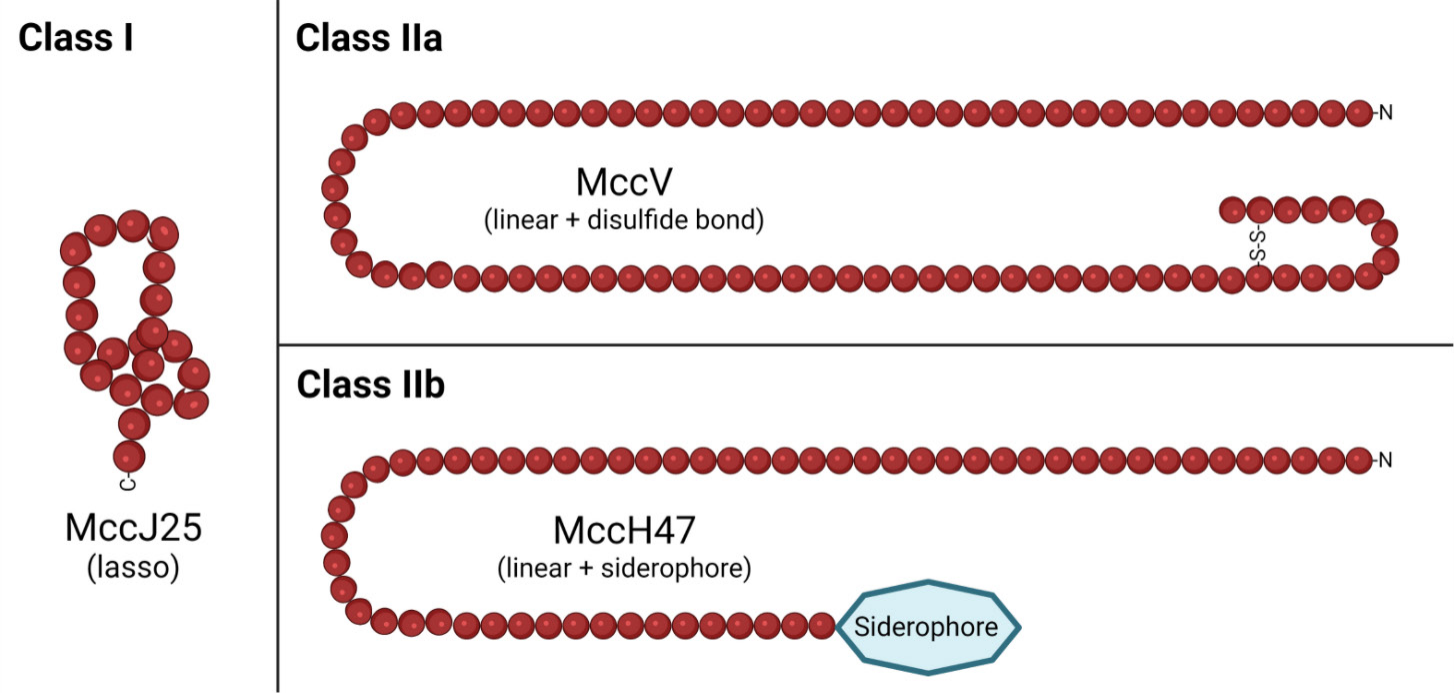
Examples of microcin structures, per class. Class I microcins (e.g. MccJ25) are heavily post-translationally modified with diverse structures. Class II microcins are larger and may have disulfide bond(s) (Class IIa, e.g. MccV) or have a C-terminal siderophore modification (Class IIb, e.g. MccH47). Mature microcin lengths are exact as represented. Created with BioRender.com.

Microcins are low-molecular-weight (<10 kDa) antibacterial peptides. They are grouped into two classes (Class I and Class II) based on broad structural characteristics. Class I microcins are small (<5 kDa) and undergo extensive post-translational modifications. Class II microcins are larger (~5–10 kDa) and are either unmodified except for disulfide bonds (Class IIa) or modified by attachment of a C-terminal iron-siderophore which facilitates uptake (Class IIb). Some examples of mature microcin structures, by class, are presented in Fig. 1. Microcins are non-lethal to the producing strain by virtue of a corresponding immunity protein or efflux system. The presence of an N-terminal signal sequence (also called the ‘leader’ peptide) with a ‘double-glycine’ cleavage site motif, found only on the Class II microcin pre-peptides, is a sequence-based differentiator between the two classes. Though commonly referred to as a ‘double-glycine’ motif, this cleavage motif can be either GG or GA [24]. Further differentiation between Class IIa and IIb microcins can also be done by sequence analysis: Class IIb microcins have a C-terminal region rich in glycine and serine residues, which is necessary for their siderophore modification [25]. Key overarching features of sequence, structure, production, export and function for each class, based on the limited number of currently known representatives, are presented in Table 1. These features will be described in more detail in subsequent sections (below). It is important to note that, due to the small sample size, general statements on microcin characteristics as presented here and elsewhere are subject to change if the number of known microcins is expanded.

**Table 1.** General characteristics of the different microcin classes

Class	Size (kDa)	Genetic location	Post-translational modifications	Microcin signal peptide	Other microcin sequence features	Immunity protein	Export machinery	Site of action
I	<5	Plasmid	Extensive, variable	Variable	None	No/maybe	Variable	Cytoplasm
IIa	~5–10	Plasmid	None (disulfide bonds possible)	Yes – N-terminal with double glycine cleavage site	None	Yes	PCAT, MFP	Inner membrane
IIb	~5–10	Chromosome	C-terminal attachment of siderophore	Yes – N-terminal with double glycine cleavage site	C-terminal serine- and glycine-rich region	yes	PCAT, MFP	Inner membrane

MFP, membrane fusion protein; PCAT, peptidase domain-containing ABC transporter.

## Diversity and prevalence

Though the microcin class was first delineated in 1976 [26], remarkably little is still known about them as a group, both functionally and ecologically. Only 15 unique microcins have been described with at least a minimum of functional confirmation and characterization, all from the *Enterobacteriaceae *. These known microcins are listed in Table 2. They include five Class I microcins, five Class IIa microcins and five Class IIb microcins. For all 15, the microcin gene sequence has been identified, with the exception of microcin D93 (MccD93), for which moderate structural and functional evidence of size and mechanism of action is available. These microcins were identified almost exclusively in *Escherichia coli *, with the exception of two Class IIb microcins found in *Klebsiella pneumoniae *, and one Class I microcin from *Salmonella enterica *. Some of the *E. coli* plasmid-borne microcins have subsequently been found in close relatives of the original source strain, for example *Salmonella* [27]. Homologues of the Class I microcins MccB17 [28–30] and MccC [31] have been identified in other species; antimicrobial activity was demonstrated for some of these. Microcin names can be abbreviated as follows: microcin V=MccV. This convention is usually followed in the recent literature, with the exception of microcin C (McC) [32].

**Table 2.** Sequence and biochemical characteristics of the 15 confirmed microcins, by class

	Original source strain	Pre-microcin length (aa)	Mature microcin length (aa)	Predicted mature microcin molecular weight (kDa)	Confirmed mature microcin mass (kDa)*	Glycine residues (%)	Hydrophobic residues (%)	Cysteine residues (SS-bonds)	Predicted mature microcin charge (pH 7)	References
**Class I**										
J25	* E. coli* Ay25 pTUC100	58	21	2.125	2.107	28.6	71.4	0	−0.99	[154]
Y	* S. enterica* pAUSMDU00010532_01	53	21	2.243	2.224	23.8	57.1	0	0.01	[116]
B17	* E. coli* pMccB17	69	43	3.255	3.074	60.5	67.4	4	−0.26	[26]
C (C7-51)	* E. coli* pMccC7	7	7	0.763	1.177	14.3	42.9	0	0.91	[21, 242]
D93†	* E. coli* LP93 pMccD93				<1				Positive	[122]
**Class IIa**										
V	* E. coli* pColV-K30	103	88	8.736		17	61.4	2 (1)	−0.13	[81]
L	* E. coli* LR05 plasmid	105	90	8.888	8.884	15.6	62.2	4 (2)	−3.36	[22]
N (24)	* E. coli* 2424 p24-2	89	74	7.222	7.224	18.9	71.6	0 (0)	2.01	[152, 243]
PDI	* E. coli* 25 plasmid	120	84‡	8.125		22.6	51.2	4 (1)§	4.84	[146]
S	* E. coli* G3/10 pSYM1	120	84||	7.957		26.2	54.8	4	5.74	[91]
**Class IIb**										
H47	* E. coli* H47	75	60	4.864	5.696	26.7	76.7	0	−0.09	[244]
I47	* E. coli* H47	77	62	6.275		12.9	50	1	−2.16	[245]
M	* E. coli* Nissle 1917	92	77	7.283	8.115	19.5	55.8	0	−1.99	[138]
G492	* K. pneumoniae* RYC492	89	74	6.598	7.342	24.3	70.3	0	−1.09	[25]
E492	* K. pneumoniae* RYC492	99	84	7.887	8.717	22.6	64.3	0	−3.99	[108, 246]

*Includes post-translational modifications, e.g. siderophores.

†Sequence is unavailable.

‡MccPDI has two cleavable signal sequences; each is 18 aa.

§One disulfide bond likely.

||MccS has 80 % pairwise identity to MccPDI and appears to have the same double signal sequence; without double signal sequences pairwise identity is 85 % – data assume double signal.

Microcins do appear to be broadly dispersed through the human gut, where they were first discovered [26], and other environments. Among human samples directly or indirectly associated with the gut, determinants for production of the currently confirmed microcins have been found in faecal *E. coli* isolates from patients with and without gastrointestinal disease [33–35], *E. coli* isolates from infections associated with gastrointestinal bacteria (e.g. bacteraemia of urinary tract origin) [36, 37], and in bacteria from the large intestinal mucosa of patients with colorectal neoplasia [38]. Microcin determinants are also found among human extraintestinal pathogenic *E. coli* (from skin/soft tissue, respiratory, intra-abdominal and genital infections) [39]. Many of these studies had sample sizes in the hundreds of isolates, with roughly half of them carrying microcin determinants and all classes of microcins (I, IIa and IIb) being well represented. Data on microcins in non-human-associated microbial communities are rare, but one study found that 36 % of colicinogenic *E. coli* isolates from the faeces of feedlot cattle and associated wastewater also carried determinants for one or more microcins. This broad dispersal of microcins and evidence that their gene clusters are horizontally transferred [40, 41], even to distantly related species such as marine cyanobacteria [42], suggests they can provide an adaptive advantage.

Growing evidence from both functional and bioinformatic studies suggests that the diversity and prevalence of microcins and related Gram-negative peptide bacteriocins is wider than currently known. We consider that the minimum criteria for confirmation as a microcin are that: (1) it is a proteinaceous antibacterial metabolite produced by a Gram-negative bacterium, (2) it is less than or approximately equal to 10 kDa in size, and (3) there is sufficient sequence or structural/functional evidence to allocate it to one of the currently defined classes (Class I or Class II); additional sequence and mechanistic information beyond this is preferred. Accordingly, various reports of microcins exist in the literature but, in our view, lack insufficient proof for this designation.

Presumptive but as yet unconfirmed microcins have been identified by screening bacterial isolates for antibiotic activity or with other functional evidence, both within and outside of the *Enterobacteriaceae *. Several suggested microcins, including one from *Proteus* sp. (* Morganellaceae *), were produced by pig gastrointestinal tract and chicken crop isolates; genetic validation and size are unavailable, but their resistance to heat is characteristic of microcins [43] (see Biochemical and Functional Features, below). Screening of *Pantoea agglomerans* (* Erwiniaceae *) strains used as biocontrol agents against the bacterial plant disease, fire blight, identified the antibacterials, Pantocin A and presumptive microcin MccEh252 [44], which are similar or identical to each other [45]. Pantocin A is <3 kDa, extensively post-translationally modified and lacks a double-glycine signal sequence [45], suggesting it may be a Class I microcin. A putative microcin from *E. coli* (Mcc1229) was recently suggested, though the class assignment is unclear [46, 47]. It lacks the signal and export features of a Class II microcin (discussed below), is larger than the Class I microcins (232 aa) and is proposed to require two immunity proteins. Another microcin-like molecule with resistance to autoclaving, pH changes and protease digestion is produced by an *E. coli* isolate from cattle faeces, though the genes encoding the toxin and its production are unidentified [48]. In the plant pathogen *Xylella fastidiosa *, expression of three adjacent MccV-like genes as well as appropriate export machinery were shown to be regulated by iron [49], as are several known microcins; these appear to have double-glycine signal sequences and no siderophore modification motif, suggesting they are Class IIa microcins. Only recently, a novel class of Gram-negative peptide bacteriocins was discovered among the *Bacteroides* using a cultural screen; they have a double-glycine signal cleavage motif but no immunity protein and differ in other ways from the canonical microcins [50]. These examples show functional evidence that additional metabolites resembling microcins exist, but suggest that a unified, systematic approach is needed for detection and confirmation. If some of these are indeed microcins, this information also indicates microcins can be found in different environments and bacterial species, and may have members with unique, undescribed characteristics.

The lack of confirmed unique microcins outside of the *Enterobacteriaceae* is surprising given that, in addition to the aforementioned cultural screens, several bioinformatic screens provide evidence of their existence. Regarding Class II microcins, N-terminal signal sequences with similar double-glycine cleavage site motifs found in association with similar export proteins seem to be widely distributed through Gram-positive as well as Gram-negative bacteria, based on bioinformatic analyses [51–53]. In fact, there are already numerous genetically, structurally and mechanistically well-characterized examples of small, Gram-positive bacteriocins with double-glycine signals that direct export (e.g. pediocin PA-1 [54] and mesentericin Y105 [55]). These signal and export protein homologies indicate such Gram-positive bacteriocins and Class II microcins may share a common ancestor [24, 56]. This potential evolutionary relationship combined with the large number of confirmed double-glycine signal-containing Gram-positive bacteriocins further supports the possibility of greater microcin diversity and prevalence. Unfortunately, these bioinformatic screens have been accompanied by little or no functional confirmation; testing for antimicrobial activity was only conducted on four Gram-positive-origin peptides which were small enough (27–29 aa) to be commercially synthesized [52]. Importantly, both Gram-positive and Gram-negative examples of double-glycine signal-containing peptides with non-toxin functions, such as roles in cell-to-cell communication [57–59] or biofilm formation [60, 61], have been identified. This necessitates functional confirmation of antimicrobial activity for candidate Class II microcins.

For Class I microcins, bioinformatics screens are more complex and specific due to the complex gene clusters needed for production and the even more limited known homologies upon which a search can be based. Nonetheless, a pipeline was developed to search specifically for lasso peptides, such as MccJ25, and it identified novel candidates among Gram-positive, Gram-negative and archaeal species [62]. One of these, from the Gram-negative freshwater bacterium *Asticcacaulis excentricus* was synthesized and shown to be active [62].

The described bioinformatics searches found evidence that predicted peptides with characteristics of microcins (e.g. double-glycine signal sequences and lasso structure) can be found among both Gram-positive and Gram-negative species. This phylogenetic dispersal of bacteriocin-like peptide sequences, in conjunction with an evolutionarily advantageous reason to possess bacteriocins (i.e. for competition, see further discussions below), supports the existence of other microcins and unknown classes of Gram-negative antimicrobial peptides. This advocates for taking advantage of shared signal sequence, export protein, structural and/or other homologies to find microcins. Exclusive of the signal sequence, there is possible weak homology between a portion of MccL and several Gram-positive bacteriocins [63]. If more microcins were discovered, perhaps additional homologies with microcin or Gram-positive bacteriocins would be discovered that could be exploited for searches.

Given the underexplored diversity of Gram-negative bacteriocins in general and microcins specifically [11, 23], combined with recent dramatic increases in the availability of bacterial deep-sequencing data, a systematic search for novel microcins could revolutionize our understanding of this bacteriocin class. Importantly, sequence repositories now contain data from numerous environmentally derived bacterial isolates, which have mostly been excluded from previous culture-based microcin screens of isolates of clinical, particularly gastrointestinal, origins. Most small-molecule antibiotics were discovered from microorganisms with an environmental origin (e.g. soil), rather than being sourced from the human niche in which the target pathogen is found. It has been theorized that bacterial species which are highly adaptable to multiple environments are equipped with a larger arsenal of bacteriocins to enable them to compete in different settings [64]. Therefore, there is no reason to overlook environmental bacteria as potential sources of novel microcins. Furthermore, sequence data allow the possibility of activity testing that is independent from the native microcin-producing environmental bacteria, which may have unknown conditions for cultivation, via heterologous production (discussed in further detail below).

Class II microcins are more amenable to such a search. Despite having low to no sequence homology in the mature peptides, the 15–18 aa signal sequences of the pre-peptides have some conserved features in addition to the double-glycine motif, and a search for common export machinery can also be incorporated. These features are not characteristic of Class I microcins. Both signal and export protein features were exploited in the only bioinformatics study specifically targeting Gram-negative double-glycine signal-containing peptides [51]. However, this study was performed almost two decades ago, when sequence availability was poor and confirmed microcins for training the search algorithm were fewer in number. Furthermore, in terms of ease of downstream utility, the absence of post-translational modifications in Class IIa microcins in particular would make them far easier to produce, study and use. Accordingly, while this review will summarize salient functional, ecological, and therapeutic features of both Class I and Class II microcins, an emphasis will be placed on Class II.

## Native production and export

Knowledge of native microcin production and export is required to assess the tractability of microcin production and/or delivery in a therapeutic context. Microcin production and export differs between Class I and Class II microcins. Due to the extensive post-translational modifications and variability in export, describing Class I production and export in general terms is not possible. Class II microcins share a more universal system of production and export, with more key features that can be described systematically. Accordingly, this review will focus on describing these features of Class II microcins; specific details of individual Class I microcin production and export have been reviewed elsewhere [65].

### Class I microcins

Among microcins, the Class I designation is a catch-all for those that are small and heavily post-translationally modified. Otherwise, there are major structural differences with limited commonalities, which are reflected in differences in production and export. MccJ25 (and its apparent homologue MccY) is a member of a class of peptides called lasso peptides, having a threaded-ring structure created by post-translational modifications (Fig. 1). It is the most well-studied Class I microcin, and its biosynthesis has been examined in detail [66–68]. MccB17 has a very different structure, where it is post-translationally modified to have four oxazole and four thiazole heterocycles to attain antimicrobial status; its structural characteristics have been recently reviewed [69]. Both MccJ25 and MccB17 use a cleavable N-terminal signal to direct export. Though the signal sequences of their pre-peptides have been identified experimentally [70, 71], neither has any particular defining features, as will be discussed for Class II microcin signals, below. The MccJ25 signal facilitates recognition and subsequent removal by the maturation enzymes [72], while the MccB17 signal is removed by an endogenous protease after post-translational modifications are complete, resulting in antibacterial activity [73]. MccC represents yet a third different Class I microcin structure. A peptide of only 7 aa, with no signal sequence, is actively taken into the target cell where it is then processed into the toxic moiety [32].

Export of MccJ25 out of the cytoplasm is accomplished by the ATP-binding cassette (ABC) transporter, McjD [70], and it exits the cell through the outer membrane factor (OMF) protein, TolC [74]. McbE, an integral membrane protein, and McbF, an ATP-binding protein, interact to export MccB17 [75]. McC is exported by the efflux pump, MccC [76]. The Class I microcins use the same proteins for both export and immunity, where immunity is achieved through efflux [32, 75, 77]. Additional immunity to MccB17 is provided by McbG [75], to MccC from a peptidase which cleaves MccC [78] and to MccJ25 from a secondary ABC transporter, YojI [79]. Notably, the export-immunity protein of MccJ25, the ABC transporter McjD, appears specific for MccJ25 [80], which contrasts with the promiscuity of Class II microcin exporters (see below).

### Class II microcins

**Fig. 2.**
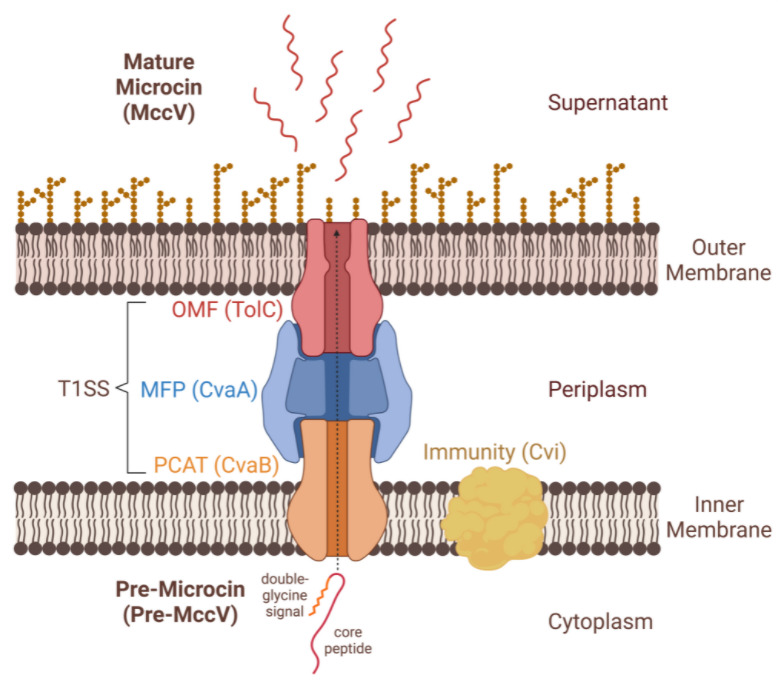
Diagram of Class II microcin secretion, using Class IIa microcin V (MccV) as an example. Secretion is accomplished via the peptidase-containing ABC transporter (PCAT), membrane fusion protein (MFP) and outer membrane factor (OMF) protein, TolC, which together create the export machinery of a non-canonical type I secretion system (T1SS). Protein names specific to the MccV system are indicated in parentheses. The N-terminal double-glycine secretion signal is recognized and cleaved from the pre-microcin by the PCAT during export. Created with BioRender.com.

For Class II microcins, the basic genetic components for microcin production and export include genes encoding the: (1) pre-microcin with a double-glycine signal sequence, (2) immunity protein, (3) C39 peptidase domain-containing ABC transporter (PCAT), (4) membrane fusion protein (MFP) and (5) OMF protein, TolC (Fig. 2). This general five-part genetic structure was first identified and described for the native microcin V (MccV, originally named colicin V) type I secretion system in *E. coli* [81–84]. In the MccV system, these key proteins are named, respectively: (1) CvaC, (2) Cvi, (3) CvaB, (4) CvaA and (5) TolC. The pre-microcin, immunity protein, PCAT and MFP are encoded in close proximity to each other, either on a plasmid (Class IIa) or on the chromosome (Class IIb). Conserved TolC is present on the chromosome of most Gram-negative bacteria and performs a variety of other non-microcin-related functions. Class IIb microcins require modification from additional gene products to obtain their C-terminal siderophore attachment, which facilitates uptake [25, 85, 86] (Fig. 1). These proteins link a derivative of endogenously produced enterobactin to the C-terminal serine of the pre-microcin [85, 86]. Bacteria with multiple microcins encoded in a microcin gene cluster can use the same PCAT and MFP to export each as well as the same genes for siderophore modification, in the case of Class IIb microcins [25].

**Fig. 3.**
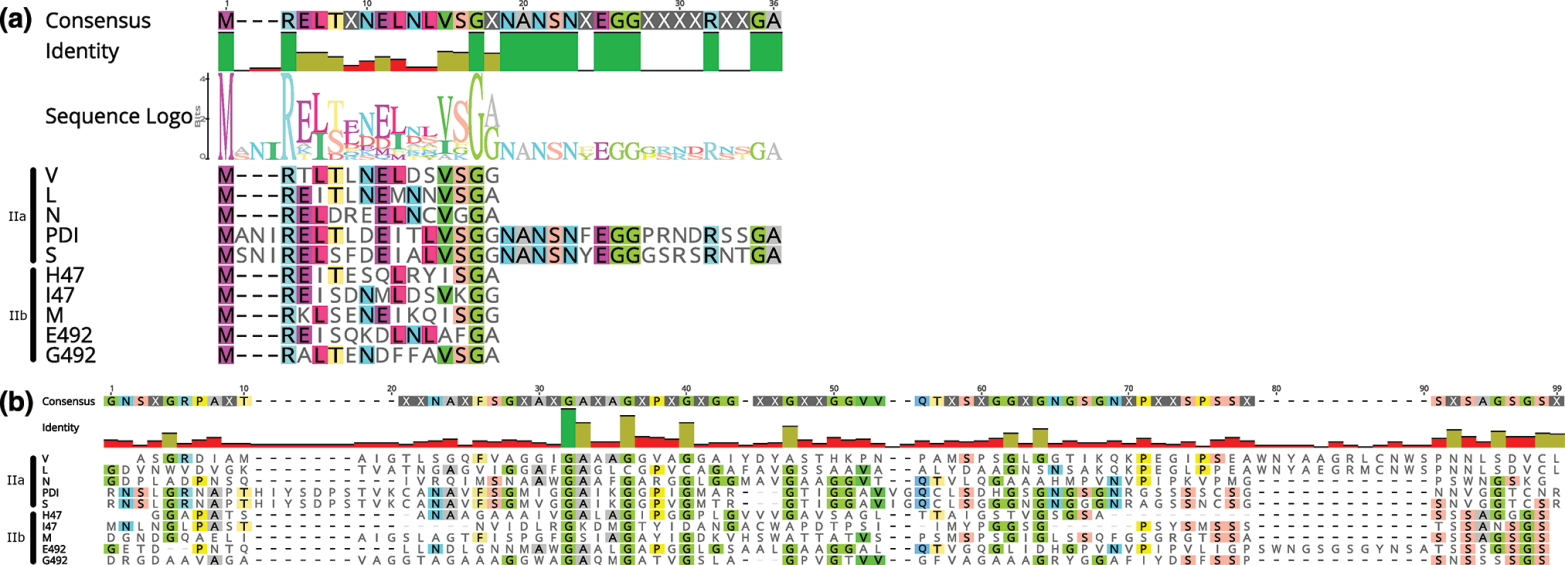
Protein sequence alignments for all 10 Class II microcins, performed and visualized in Geneious Prime 2021.1.1. Class IIa microcins (V, L, N, PDI and S) are shown at the top of the alignments, and Class IIb microcins (H47, I47, M, E492 and G492) are shown at the bottom, as indicated. (**a**) Alignment of the N-terminal double-glycine signal sequences. The consensus sequence (generated with a 50 % identity threshold), sequence logo and percentage identity per residue position are shown above the alignment. MccPDI, and probably MccS, has a second 18 aa signal which is shown here following the first signal. (**b**) Alignment of the core peptides (without the signal sequences). The consensus sequence (50 % identity threshold) and percentage identity per residue position are shown above the alignment. The conserved siderophore motif of the Class IIb microcins, consisting of eight glycine- and serine-rich residues, is evident at the C-terminal portion.

The pre-microcin contains the N-terminal signal sequence of 15–18 aa terminating in a double-glycine cleavage site motif of GG or GA; this signal sequence is cleaved after the second residue in the motif (either G or A, respectively) during export. In the case of MccPDI, there are two back-to-back double-glycine signal sequences, each of 18 aa, which are both cleaved in succession [87]. Both signals are necessary for antibacterial activity, though their exact function in secretion and/or activity is unclear. This may also be the case for MccS, which appears to have the same signal sequence duplication and shares 80 % pairwise identity with the MccPDI pre-microcin (by pairwise blast of protein sequences [88]). In addition to the cleavage site, Class II microcin signal sequences share additional sequence similarity, as seen in an alignment and sequence logo (Fig. 3a). The microcin structural component, or core peptide, which follows the signal sequence has no obvious defining features, and most of those from the 10 known Class II microcins have very low pairwise identity (Fig. 3b). Their core peptides range in size from 60 to 90 aa (MccH47 to MccL, respectively).

Each Class II microcin has an associated self-immunity protein, which is typically encoded immediately adjacent to the pre-microcin gene. The immunity protein is requisite for successful microcin secretion, otherwise the microcin producer is inhibited or killed [89, 90]. The ORFs of the pre-microcin and immunity protein often overlap, further indicating their need for tight co-regulation. It is not currently possible to locate the immunity proteins based on any conserved sequence features. Immunity candidates are identified based on proximity to the microcin and small size, though they must be confirmed experimentally. Immunity proteins range in size from 51 to 216 aa [63, 91] (median 82 aa), with the largest, the immunity protein for MccS, being an outlier in size. Predictions of transmembrane helices and localization to the inner membrane are also characteristic of an immunity protein, reflecting a position where they can interact with or physically block the microcin in some way to prevent cell entry. Immunity proteins are typically highly specific to their cognate microcins.

Class II microcin processing and secretion is initiated upon recognition of the pre-microcin secretion signal by the C39 peptidase domain of the PCAT. The C39 peptidase domain family has calcium-dependent cysteine peptidase activity, which is conferred by three key residues (on CvaB of the MccV system: Cys32, His105, Asp121) which make up a catalytic triad [92, 93]. This triad is conserved across all Class II microcin PCATs (based on sequence alignments using Geneious Prime 2021.1.1, https://www.geneious.com, data not shown). The signal sequence is cleaved from the pre-microcin after the double-glycine motif via the cysteine peptidase activity, concurrent with export. These residues are a key differentiator from the highly homologous C39-like peptidase domain of other ABC transporters, which lacks the requisite peptidase activity to cleave the signal sequence [94].

Secretion is enabled by coupling of the PCAT, MFP and TolC to create a non-canonical type I secretion system (T1SS), which secretes peptides directly to the supernatant without a periplasmic intermediate [95] (Fig. 2). In the classic T1SS, the secreted protein (e.g. haemolysin A) instead has a C-terminal secretion signal which is not cleaved [96]. Tripartite efflux systems are common in Gram-negative bacteria. MFPs connect a primary active transporter located on the inner membrane to a protein of the OMF family, which provides a conduit for export. The transporter and MFP form a cognate pair with some substrate specificity, while the OMF (e.g. TolC) is more promiscuous and capable of partnering with different secretion systems to efflux different types of substrates.

Details of the specific mechanics of microcin secretion have not been fully described, but related studies in Gram-positive bacteria provide some clues. Gram-positive bacteria also contain PCATs, which both cleave and export double-glycine signal sequence-containing peptide substrates through the single membrane without additional protein partners [56, 97, 98]. These substrates include many, but not all, of the Gram-positive Class IIa, IIb and IId bacteriocins. The first PCAT crystal structure was recently determined for PCAT1 of *Clostridium thermocellum *, which cleaves the double-glycine motif signal sequence of the putative substrate [99]. The PCAT1 transmembrane tunnel is a large, alpha-helical barrel-shaped cavity. Collective structural findings support the idea of alternating tunnel access, where the N-terminal signal sequence is bound to the peptidase domain, allowing the C-terminal substrate to position into the cavity prior to cleavage; after cleavage the cytoplasmic entrance closes and the tunnel exit opens [99]. The size of the transmembrane cavity limits cargo size in this model. If Class II microcin PCATs function similarly, an upper size limit for microcins would be expected based on the PCAT cavity size. Currently, the largest confirmed mature microcin cargo is MccL at 90 aa and 8.8 kDa [63].

## Uptake and mechanisms of action

The ways microcins get across membranes are varied, and mechanistic knowledge about uptake of the limited number of microcin examples is incomplete. However, despite key structural differences that distinguish microcin classes, there are some repeated themes among uptake of Class I, Class IIa and Class IIb microcins. First, microcins must engage with an ‘outer membrane receptor’ to allow them to cross the outer membrane. Normally, outer membrane receptors allow essential nutrients that are too large to passively diffuse through small porins, such as iron-containing complexes like siderophores, to cross the membrane in an active, TonB-dependent manner [100]. Microcins can utilize some of these same receptors for uptake. They may even mimic the provision of desirable nutrients to achieve uptake (e.g. siderophore attachment used by Class IIb). Several microcins have therefore been described as ‘Trojan horse’ antibiotics [101, 102], due to their ability to trick cells into uptake prior to rendering their toxic effects. In fact, the Trojan horse concept was originally described in relation to iron uptake [103].

Accordingly, across these microcin classes, the same or similar types of outer membrane receptors may be used for uptake (Table 3), and all currently identified receptors are influenced by nutrient limitation. These receptors are predominantly involved in iron uptake (Cir, FepA, Fiu, FhuA). The influence of OmpF is increased during low osmolarity (nutrient-limited) conditions [104], for example glucose limitation [105]. Energy to transport the microcin can be provided by the TonB system in the case of iron uptake receptors [106–108]. Other TonB-dependent outer membrane receptors have been identified that are involved in uptake of nutrients besides iron, such as vitamin B12 (also used for uptake of some colicins) [109, 110], zinc [111], nickel [112] and sugars (e.g. maltodextrins [113]). It seems possible that microcins could make use of some of these or other as yet undiscovered routes for uptake as well, which would be useful information for therapeutic applications [114].

**Table 3.** Features of uptake and mechanism of action for the 15 partially characterized microcins Features that have been analysed but ruled out are indicated in parentheses. Features that have not been analysed are indicated as ‘unknown’.

Microcin	Outer membrane receptor	Uptake	Inner membrane receptor/target*	Mechanism	References
**Class I**					
J25*	FhuA	TonB-ExbBD	SbmA	Crosses inner membrane to obstruct RNA polymerase secondary channel (inhibits transcription); inhibits respiratory chain enzymes and increases production of reactive oxygen species	[107, 115, 120, 123, 247, 248]
Y	FhuA	Unknown	SbmA	Unknown	[116]
B17	OmpF	Unknown	SbmA	Crosses inner membrane to inhibit DNA gyrase	[117, 121]
C (C7-51)	OmpF†	Unknown	YejABEF	Blocks protein synthesis at translational level by targeting aspartyl-tRNA synthetase	[65, 118, 249]
D93	Unknown	Unknown	Unknown	Inhibits DNA biosynthesis	[122]
**Class IIa**					
V	Cir	TonB-ExbBD	SdaC	Disrupts inner membrane potential; pore formation not demonstrated	[89, 106, 250]
L	Cir	TonB-ExbBD OR -TolQR	(NOT SdaC, ManYZ)	Disrupts inner membrane potential; no permeabilization; depolarization to access unknown target	[127]
N (24)	(NOT FepA, Fiu, Cir)	Unknown	Unknown	Unknown	[125]
PDI	OmpF, possibly other(s)	Unknown	(NOT SbmA, YejAVEF, ManYZ)	Two microcin products form multimers which permeabilize inner membrane, leading to cell death	[87, 232]
S	Unknown	Unknown	Unknown	Unknown	[91]
**Class IIb**					
H47	Cir, FepA, Fiu/IroN‡	TonB	AtpBEF	Targets F_0_ proton channel of ATP synthase, which is located on inner membrane	[138, 251, 252]
I47	Cir, FepA, Fiu	Unknown	Unknown	Unknown	[245]
M	Cir, FepA, Fiu/IroN‡	TonB	Unknown	Unknown	[138]
G492	Unknown	Unknown	Unknown	Unknown	[25]
E492	Cir, FepA, Fiu/IroN‡	TonB	ManYZ	Inserts into inner membrane; destabilizes membrane potential by pore formation; causes depolarization and permeabilization	[85, 90, 108, 138, 253]

*As discussed in the text, the line between inner membrane receptor versus target can be indistinct, so inner membrane proteins appearing to fit part of either description are listed here.

†From a citation of unpublished data; experimental evidence is not shown [65].

‡Fiu is a siderophore receptor of *Escherichia coli*; IroN is a siderophore receptor of *Salmonella enterica*.

A second protein or protein complex on the inner membrane can be required to allow the microcin to interact with or cross the membrane, resulting in destructive effects on the inner membrane and/or providing access to a cytoplasmic target [89, 90, 107]. This protein can be referred to as an ‘inner membrane receptor’ in cases where it is known to facilitate access to the target, but is not itself the target, as for Class I microcins (see below) and MccV [89]. However, in many cases this delineation between receptor and target is unclear due to lack of mechanistic details.

### Class I microcins

Despite their varied and complex, post-translationally modified structures, Class I microcin methods of uptake and mechanisms of action have been more thoroughly examined than for Class II microcins (Table 3). Many of these features have been reviewed [65], but will be described briefly here. Uptake is achieved through the FhuA [115, 116] or OmpF [65, 117] receptors. For the only Class I microcin where the energetic means of uptake is known, MccJ25, the TonB-ExbBD system is used [107]. Identified inner membrane receptors are the transporters, SbmA [107, 116, 117] or YejABEF (ABC transporter) [118]. SbmA belongs to a proposed new class of secondary transporter [119]. It is involved in the uptake of diverse peptide substrates such as the mammalian antimicrobial peptide (AMP), Bac7, and the molecular mechanism of substrate translocation, including a key residue involved in initial microcin binding, has been proposed [119]. The cytotoxic targets of Class I microcins are varied aspects of nucleic acid and protein synthesis, which contrasts with the currently known inner membrane-active mechanisms of the Class II microcins. Class I microcins have been shown to specifically inhibit RNA polymerase (MccJ25) [120], DNA gyrase (MccB17) [121] and aspartyl-tRNA synthetase (MccC) [21], with general inhibition of DNA biosynthesis indicated for MccD93 [122]. MccJ25 has a second cytotoxic effect: it inhibits respiratory chain enzymes and increases the production of reactive oxygen species [123].

### Class II microcins

Characterization of target cell uptake and mechanisms of action for Class II microcins is limited and incomplete (Table 3). For three of the 10 known Class II microcins, virtually nothing has yet been confirmed in this regard. Class IIa microcin mechanisms of action are more poorly studied than for Class IIb ([124], Table 3). For microcins where some features of uptake are known, they are first recognized by a specific outer membrane receptor (iron-siderophore receptors Cir, FepA, and/or Fiu/IroN, or porin OmpF). The receptor(s) for MccN are unknown but are not these siderophore receptors [125]. Potential receptors for MccS and MccG492 have not been tested, but given sequence similarity to MccPDI and Class IIb siderophore microcins, respectively, one could speculate they may use OmpF and the iron-siderophore receptors, respectively. For the Class IIb microcins, their siderophore attachment is not always necessary for antimicrobial activity but at a minimum significantly enhances it; unmodified MccE492 is still exogenously bactericidal [85, 90], but its activity is increased up to eight-fold upon modification, and the range of species it is active against is expanded [85]. The process by which microcins encounter and engage with the outer membrane receptor has not been discussed, with the exception of MccPDI. A model for MccPDI uptake proposes that two cleaved forms, resulting from separate cleavage events for the two signal sequences, interact separately with OmpF for entry and subsequently join in the bioactive multimer upon reaching the periplasm [87]. When the mechanism for uptake is known, it has been shown to occur via the proton-motive force of the TonB-ExbB-ExbD inner membrane complex [106, 108].

After crossing the outer membrane and entering the periplasm, Class II microcins interact with an inner membrane receptor/target, unless the appropriate immunity protein is present on the inner membrane. MccV and MccE492 are the only Class IIa and IIb microcins, respectively, for which the inner membrane receptors (SdaC and ManYZ, respectively) have been confirmed. The microcin usually targets the inner membrane, and sometimes inserts into it. The interaction with the inner membrane, facilitated by the receptor, allows the microcin to disrupt inner membrane potential which can lead to depolarization and/or cause pore formation which can lead to permeabilization. In the case of MccE492, the core peptide (without the signal sequence) is toxic in the cytoplasm, suggesting that MccE492 needs to cross the inner membrane and insert into it through the cytoplasmic side [90]. There is some evidence that unmodified MccH47 may also be cytoplasmically active in the absence of its immunity protein [86, 126]. The major mechanistic novelty among Class II microcins is MccH47, which specifically targets the F_0_ proton channel of ATP synthase (spans the inner membrane). However, it is possible that the commonly observed microcin-induced depolarization and permeabilization may function, at least in some cases, to provide access to a specific unknown target which is the ultimate cause of cell death [127].

### Comparisons with other bacteriocins

Many of the themes for microcin uptake and activity are also broadly characteristic of colicins and, to some extent, Gram-positive bacteriocins (particularly double-glycine signal-containing bacteriocins, as discussed earlier). For example, many colicins use nutrient receptors (e.g. siderophore receptors Cir, FepA and FhuA; vitamin B12 receptor BtuB) for uptake and TonB for translocation, allowing activity on the inner membrane; a review of colicin import summarizes the key features [110]. Also, most colicins can be broadly divided into two groups based on their cytotoxic effects: enzymatic (nuclease) colicins and pore-forming colicins, some of which act as ionophores [110]. Similarly, the majority of Class I microcins inhibit nucleic acid synthesis in some way, while Class II microcins tend to form pores or at least depolarize the inner membrane as part of their mode of action. Recent advances in determining the specific interactions with membrane components that allow colicins to cross the outer membrane [128–130] as well as the inner membrane [131] could shed light on the mechanisms used by microcins, or at least provide clues as to how to address these questions for microcins.

Due to the lack of an outer membrane, fewer obvious similarities exist for uptake and mechanisms of action for Gram-positive bacteriocins. However, a notable example exists in the targeting of the mannose phosphotransferase system (man-PTS). MccE492 utilizes the ManYZ complex as an inner membrane receptor [90], which is homologous to the ManCD complex used by some Gram-positive Class IIa and IId bacteriocins for both uptake and activity [132]. One could presume there are other features in common between the Gram-negative inner membrane and the Gram-positive membrane that microcins may act upon.

The possibility of shared evolutionary history between ostensibly disparate classes of bacteriocins is worth considering. Perhaps these bacteriocins are convergently taking advantage of a key suite of uptake and action routes that are available for exploitation. It is particularly curious that both peptidic and proteinaceous Gram-negative bacteriocins target similar host features given their very different sizes. A better understanding of how nature allows these macromolecules to slip past the ‘impermeable’ (i.e. minimally and selectively permeable) Gram-negative outer membrane [133] may inform researchers’ attempts to get around the largest barrier to antimicrobial delivery.

## Regulation

Unlike for colicins, where induction of production by stress factors such as DNA damage and nutrient starvation has been well documented [134–136], the exact factors triggering the initiation of microcin production are less clear and vary more per microcin. Similar to colicins, there is a demonstrated association with nutrient limitation, as mentioned above, which relates to the involvement of nutrient receptors in microcin uptake into target cells. In particular, iron has been demonstrated to regulate microcin production [106, 137, 138]. Low iron concentrations drive an increase in microcin production as well as an increase in iron uptake receptors on the target cells, which are the most commonly identified type of receptors utilized by microcins thus far. For MccPDI, which uses a non-iron uptake receptor, OmpF, low osmolarity conditions induce MccPDI expression, which is regulated by the two-component system EnvZ/OmpR [87]. There is also an association with growth phase of the producing bacteria, though this should be correlated to nutrient availability. Native production of the Class I microcins and MccPDI is upregulated during the stationary growth phase [139, 140], presumably when nutrient depletion is sensed. However, active MccE492 is only produced during exponential growth [141], which is also the phase when production of MccN peaks [125]. Gram-positive bacteriocin production can be regulated by quorum sensing peptides [142, 143], which has now also been demonstrated for MccPDI [144]. Additional explorations to determine if/how quorum sensing or other types of mechanisms regulate other microcins are needed.

## Spectrum of activity

Though bacteriocins and microcins are usually described as having activity only against close relatives, many defy this generality [145]. For example, MccL, produced by *E. coli *, is active against *Pseudomonas aeruginosa* [63], which is not even within the same order (* Enterobacterales* versus *Pseudomonadales *, respectively). In addition to *Salmonella *, *Shigella* and *Pseudomonas *, the most recently discovered Class I microcin, microcin Y, is active against some strains of *Bacillus* and *Staphylococcus *, both Gram-positive genera [116]. The ability of specific microcins to inhibit particular human pathogens [146, 147], including multidrug-resistant strains encoding carbapenemases and extended-spectrum beta-lactamases [148] and foodborne strains [149, 150], has been demonstrated in many cases. Therefore, there is no reason to pre-emptively exclude the possibility of finding novel microcins with activity against unrelated species or particularly resistant/pathogenic strains.

For microcins that have been successfully purified, their activity falls in the nanomolar to micromolar range. MccN, MccL, MccJ25 and E492 have MICs for their primary target strains in the nanomolar range (4–150 nM) [63, 85, 151, 152], while MccH47 has MICs in the low micromolar (1–12 µM) range [148]. In a side-by-side comparison, the MIC of MccJ25 for *Salmonella* Newport was 30 nM, far more potent than the Gram-positive bacteriocin, reuterin, or the small molecule antibiotic, rifampicin [153], suggesting the possibility of microcins as an antibiotic alternative. However, high relative potency could be biasing the pool of currently known microcins by making them easier to detect; the existence of weaker microcins could partially explain the difference between computationally suggested versus experimentally confirmed microcins.

## Biochemical and functional features

Microcins have many characteristics that make them promising for antimicrobial peptide applications. They are generally considered [23] to be resistant to heat [63, 125, 154], proteases [125, 155] and extreme pH [154], imparting a level of stability that is not often seen for peptides. Microcins are also typically non-toxic towards human cells. However, these concepts have not been rigorously and systematically validated across microcins. For example, protease sensitivity varies by protease, and the selection of proteases tested varies widely [63, 125].

Microcin core peptides have diverse primary sequences, but they do share some global properties. Some biochemical features of known microcins (*n*=15) are summarized in Table 2. Experimentally confirmed microcin masses, which reflect any post-translational modifications, range from 1.2 to 8.9 kDa. Microcins are glycine-rich, with the glycine residues relatively dispersed through the length of the mature peptide. Amino acids have been defined as abundant at compositions ~8 % or greater [156]. The average glycine composition of the predicted or confirmed mature microcins (excluding post-translational modifications) is 23.8 %. Though glycine is often found at a higher frequency in AMPs generally [156], microcin glycine content is particularly high. For comparison, a search of naturally occurring AMPs in APD3 (the antimicrobial peptide database, version 3 [9]) indicates Gly content is 14.8 % for anti-Gram-negative AMPs from all sources (*n*=316) and 17.5 % for pediocin-like Class IIa bacteriocins from Gram-positive bacteria (*n*=17). One could speculate that this may allow increased flexibility to facilitate crossing of the outer and inner bacterial membranes. Microcins are also highly hydrophobic (average of 61.2 % hydrophobic residues), which would help them interact with target cell membranes as well, either for crossing them (outer and/or inner membrane) or insertion (inner membrane, depending on the mechanism of action). Most microcins have a neutral or anionic charge (except Class IIa microcins N, PDI and S). They do not require a cationic charge to disrupt the outer membrane like many AMPs do because they use specific receptors to cross the membrane.

The existence of microcin-specific immunity proteins or efflux systems is a useful feature which can be used to avoid microcin toxicity toward the producing cell. As with all antimicrobials, the likelihood of developing resistance to microcins should be contemplated. Their increased target specificity compared to broader range antimicrobials probably reduces collateral damage across a microbial community, which should decrease the development of resistance [157, 158]. Though microcin production and target cell uptake are often triggered by nutrient limitation, mutation/elimination of nutrient acquisition mechanisms to prevent microcin uptake would not be advantageous to the target cell. Combinatorial antibiotic therapies incorporating bacteriocins may help prevent resistance, with a possible added benefit: bacteriocin–antimicrobial synergy has been demonstrated in a number of cases [158, 159]. Production of multiple microcins, which often occurs in nature, should help in a similar fashion if the microcins exploit different receptors [160]. Collectively, these characteristics have potential to overcome the major impediments to use routinely cited for antimicrobial peptides, such as lack of target specificity, mammalian cell toxicity and degradability [161].

Purified microcin is needed for a thorough characterization of antibacterial activities and physiochemical properties. However, microcin purification, especially at large scale, can be challenging [152]. Chemical synthesis is generally not possible due to the complexity of post-translational modifications or simply microcin length. Large-scale synthesis of high-purity linear peptides >30 aa long is costly. For these reasons, microcins are routinely obtained from bacteria. Purification is performed using the native producer or a heterologous strain carrying the genes necessary for microcin synthesis, immunity, export and modification as needed [22, 125, 152, 162–165]. Typically, the supernatant of the secreting strain is pre-purified by column chromatography, followed by HPLC [22, 125, 152, 165]. Low nutrient medium is often sufficient for production, but a better understanding of the environmental signals that induce microcin expression may help increase the yield that can be obtained. Investigators sought to increase MccN production by using a fusion protein to increase solubility of the hydrophobic core peptide, but the final cleaved peptide was inactive [152]. Other production attempts using yeast or lactic acid bacteria expression systems were unsuccessful [152]. The development of methods for large-scale microcin purification will aid in their characterization and is necessary for industrial application.

## Ecological roles of microcins

The ecological roles of microcins are not well elucidated, as too few ecologically relevant studies of microcin function have been conducted. Most studies are *in vitro* examinations of a single microcin-producing strain or purified microcin with a single target strain per assay, designed with the goal of demonstrating antimicrobial activity [91, 116, 146]. *In vitro* explorations of *in vivo* phenomena can be misleading; just because a bacteriocin is antimicrobial in high concentrations *in vitro* does not mean it has the same ecological function [12, 166]. Microbiome-based studies provide an essential new avenue to explore community effects of bacteriocins. These can be conducted either *in vitro* or *in vivo*, but methodologies are still rapidly developing and even the best-designed experiments cannot fully replicate naturally occurring conditions of interest. If a producing strain is engineered to constitutively express the microcin, regardless of condition, this can obscure its natural function.

### Competition

The natural ecological role of bacteriocins, including microcins, is broadly understood to be in competition with other bacteria. The intricacies of the role of bacteriocin production in competition and associated ecological and evolutionary theory has been well reviewed previously [8, 166–168], as it has for microcin production [64], based on a smaller body of literature. Because of the human health implications, there has been a focus on exploring the roles of bacteriocins and microcins in the gut, where they are widespread [169]. Though available data showing microcinogenic bacteria can outcompete other strains in the mammalian gut *in vivo* are certainly suggestive [170], there is insufficient evidence to fully support a general role in competition or completely clarify the function(s) of individual microcins. Ecological experiments with colicins indicate that community composition, spatial structure, bacteriocin strength and other environmental cues determine when and how they are deployed in competition and what their resulting effects are on the microbial community [135, 171–175]. Similar experiments with microcins, where one or more microcins are pitted directly against each other, would be useful to begin to characterize and contextualize their role in competition.

Competition can promote microbial diversity rather than reduce it [167, 172, 175], which in the case of the human gut microbiome is associated with increased stability [176, 177]. In fact, weak bacteriocins may be favoured in the environment to help maintain balance rather than obliterate a specific opponent [172, 178], according to the ecological competition phenomenon of the ‘survival of the weakest’ [179]. Particularly when comparing microcins to colicins, which are both produced by enterobacterial gut inhabitants, it is tempting to speculate that microcins serve exactly this purpose and thus may be much more widely distributed than has been recognized. A recent competition experiment between three Gram-negative bacteriocin producers further demonstrates this possibility [180]. Strains producing colicin E3 (inhibits protein synthesis), colicin E7 (degrades DNA) and microcin V (disrupts the cell membrane) were pitted against each other in a variety of competition scenarios. In paired interactions, MccV was demonstrated to be the weakest toxin. However, in competitions among all three strains, MccV was able to dominate in the majority of scenarios tested, while the producer of the strongest toxin (colicin E3) was never dominant. However, this did result in an unbalanced community, contrary to results from the previously mentioned work [173]. The authors theorize that the disadvantage of being the strongest toxin may place an upper limit on evolving towards more potent toxins and explain the relative frequencies of different mechanisms of action [180].

Since many microorganisms have systems in place to detect DNA damage, nuclease colicins with DNA-degrading activity can cause dramatic effects in a community, which can affect the producer negatively due to retaliation by competitors [171]. In addition, colicin release burdens the producing populations because producers must lyse to release them, so they are quite costly traits. Microcins are much less extreme in these regards, as they can create more subtle effects and do not cause producer death. For example, in the classic assay to detect bacteriocin production, MccS does not produce a zone of inhibition, but it can suppress adherence of enteropathogenic *E. coli* to intestinal epithelial cells [91]. Incidentally, microcins tend to co-occur alongside other microcins or colicins within the same isolate [181, 182], and multiple bacteriocin production is common in general. This suggests that being able to produce multiple bacteriocins with different, complementary uses is beneficial. Certainly, microcins and colicins represent two different approaches to bacterial competition and are probably deployed under different ecological circumstances.

### Regulation of microbial communities

Regardless of if/when microcins function in an actively competitive manner, they do regulate their surrounding microbial communities. Effects on community structure and composition can be shown and perhaps harnessed to therapeutic benefit. Experimental evidence demonstrating the ability of microcins to regulate and control individual microbes or groups of microbes within microbial communities is growing [150, 170, 183–185]. That known microcins originated from gut bacteria conveniently dovetails with the fact that the gut is the foundational site of microbiome studies and continues to dominate this field. A recent review on the interplay between microcins and the gut ecosystem describes the origins of this work and its implications in further detail [64].

Results on the effects of microcins in the gut have been obtained using two different formats for microcin application: *in situ* microbe-based microcin production versus treatment with purified microcin. While there are pros and cons to each regarding what can be learnt about microcins, both types of studies are directly relevant to potential applications via probiotic microbiome modulators or standalone drugs, respectively (see subsequent sections on probiotics and therapeutics). Among current studies, which are few in number, testing of microbial community effects has been done for several of the currently known microcins in varied animal models (murine, porcine, avian) as discussed in detail below. Effects have been measured for individual pathogens, key community taxa and the microbiome as a whole.

Microcin production by engineered or natively secreting strains *in vivo* provides the chance to observe a scenario closer to what occurs in the natural production of microcins and can address some aspects of their role in competition. Here, microcin production is tied to the characteristics of the producing strain, which can be beneficial or requisite to the desired effect (e.g. if the effects of the microcin are tied to the colonization capabilities of the producer). In the first significant study of microcin activity *in vivo*, performed more than two decades ago, an avirulent avian *E. coli* strain transformed with a plasmid encoding production of Class IIa microcin N significantly reduced *Salmonella typhimurium* colonization in the chicken intestinal tract when supplied in drinking water [150]. This effect was not due to successful colonization by the microcin-secreting strain, so was only observable if administration was continuous rather than dose-based.

Much more recently, the effects of native microcin production *in vivo* by the probiotic *E. coli* strain Nissle (this strain is discussed in further detail in sections below) was rigorously examined in a mouse model [170]. The Class IIb microcins of this strain, MccM and MccH47 [25], do indeed allow it to outcompete and inhibit enterobacterial pathogens in the gut, but only when the gut is inflamed (specifically, in a mouse colitis model) [170]. Iron limitation (the natural condition of the gut) was necessary to prompt native microcin production in this study. This demonstrates a potential downside for natively regulated microcin production; microcins could not be produced by *E. coli* Nissle at sites where iron depletion is infeasible or detrimental. Also, the roles of MccM versus MccH47 cannot be parsed in this case, where both microcins are natively regulated at the same time in the same strain.

Encouragingly, this *in vivo* mouse study found that treatment with *E. coli* Nissle did not significantly alter the rest of the gut microbiome (i.e. other than the targeted *E. coli *), with the caveat that the colitis mouse model has lower microbial diversity than a healthy gut [170]. One additional study involving microcinogenic bacteria also tried to address the extent to which collateral modulation of the microbiome may or may not occur with bacteriocin treatment. Treatment of piglets with a cocktail of probiotic *E. coli* strains which secrete both colicins and microcins (MccV, MccB17) in advance of a challenge with enterotoxigencic *E. coli* (ETEC) resulted in significant reductions in pathogen counts and the duration of diarrhoea [186]. There were no significant differences in microbial diversity among the treated versus untreated piglets [186], suggesting the probiotic strains affected pathogenic strains without obvious impacts on the rest of the microbiome.

Studies demonstrating regulation of microbial communities using purified microcin have emerged only in the last few years, probably due to improvements in the purification process. The majority of these have examined purified MccJ25 treatment [184, 187–191], though one study used purified MccC [185]. With MccJ25, there has been an emphasis on investigating its anti-inflammatory properties and improvements in intestinal barrier function, which are presumably due, at least in part, to effects on the microbial community and/or elimination of specific pathogens [184, 187, 190, 191]. Several of these studies have found increases in the abundance of specific taxonomic groups (e.g. *Bifidobacterium* and *Lactobacillus *) [188–190] that are described as probiotic and correlate with mammalian gut health [192], concurrent with decreases in faecal *E. coli* or total coliforms (i.e. undesirable taxa) after MccJ25 treatment.

Collectively, though few in number, these *in vivo* studies show that microcins can regulate microbial communities in a selective manner and so may be useful in targeting specific pathogens. Surprisingly, there has not been much attempt to characterize the effects of microcins on microbial communities *in vitro*; *in vitro* studies have focused on effects on individual microbes only. Perhaps *in vitro* studies could be a simpler and more direct approach for initial systematic screening of microcin effects on a community, prior to implementation in an animal model, where numerous other biological factors must be taken into account. An outstanding question is how the effects of microcins on a community may differ depending on their specific mechanisms of action. A uniform approach for multiple, different microcins is needed to begin to address this question.

### Additional roles

There are indications that the natural roles of microcins may include other features which extend beyond or work in conjunction with competition. For MccB17 and putative Mcc1229, their ability to promote Shiga toxin production in pathogenic strains when produced by commensal strains demonstrates an unanticipated regulatory effect on the associated microbial community that may be important in the clinical manifestation of disease [46, 193]. A curious feature has been observed for MccE492, where it aggregates to form amyloid fibrils during the stationary phase of the producing cells, resulting in the loss of cytotoxic activity [194]. These amyloids behave in a prion-like fashion, where they can be maintained in subsequent generations of cells, ostensibly as a means to regulate protein function [195]. MccB17 has possible host immune-modulatory effects; fragments of MccB17 containing only an oxazole heterocycle can trigger intestinal inflammation associated with inflammatory bowel disease, and this effect is independent of MccB17 antimicrobial activity [196]. These examples demonstrate that microcins can have a wide variety of functions and characteristics, with additional roles yet to be discovered.

## Microcin production as a probiotic and/or pathogenic trait

Microcin production is a trait frequently found both in strains described as probiotic [43, 91, 138, 197, 198] as well as those described as pathogenic [27, 33, 37, 39, 199]. Given that a bacterial species can function as either a probiotic or a pathogen in different contexts, and these designations can be subject to interpretation, the role of microcinogeny is not definite. What successful probiotics and pathogens have in common, however, is an ability to colonize the host, and microcins are probably able to contribute in this regard.

As interest has grown in preventing or treating microbial dysbiosis through microbiome modulation [200], probiotics have gained in prominence as a possible therapeutic option. Probiotics are ‘live microorganisms which when administered in adequate amounts confer a health benefit on the host’ [201]. In practice, most probiotic bacterial strains have been identified based on their ability to colonize and benefit the gastrointestinal tract, where the majority of microbiome research has been conducted thus far. *E. coli* is the most frequently identified Gram-negative species having probiotic strains, and *E. coli* Nissle is the most widely examined [202, 203], with a demonstrated ability to inhibit pathogen colonization in the gastrointestinal tract [170, 204, 205].

Bacteriocins are frequently encoded by bacterial strains identified as probiotic, and bacteriocinogeny is a recognized mechanism for microbiome modulation by probiotic strains [206]. Bacteriocins can help their host bacterium to function in both probiotic and antibiotic capacities, which can generally facilitate colonization/competitive exclusion or more targeted inhibition/killing effects, respectively [207, 208]. For many years, these functions were largely theoretical, but confirmatory experimental evidence has become more prevalent. For example, mice inoculated with *E. coli* strains differing only in production of different colicins indicated colicin-producing strains colonized the mouse gastrointestinal tract at higher densities over a longer time period (>3 months) than the colicin-negative control [209]. Additionally, some evidence suggests that some microcins may have evolved to benefit the health of the host. When a suite of microcins was tested for activity against both commensal and pathogenic *E. coli* strains from piglets, susceptibility to most microcins was significantly higher for the pathogenic strains [186].

Microcins, specifically, are often associated with probiotic strains. Two of the most well-known and widely used probiotic drugs on the market, Mutaflor and Symbioflor 2, contain microcin-producing strains of *E. coli *. Mutaflor is composed of *E. coli* Nissle, which secretes the Class IIb microcins M and H47 [25], while Symbioflor 2 contains *E. coli* G3/10, which produces the Class IIa microcin S [91]. In fact, microcins M and S are named after their respective drugs, which are both indicated for several gastrointestinal conditions (e.g. irritable bowel syndrome). *E. coli* H22, also suggested for probiotic use in livestock and humans, produces microcin C, in addition to other bacteriocins [210].

Given that colonization is a key requisite probiotic feature [211], it stands to reason that other colonization-promoting features may coincide with bacteriocin production. This seems to be the case for type I pili, which facilitate adhesion and biofilm formation. *E. coli* strains encoding *fimA* (*pilA*), the major pilin subunit, are significantly more likely to encode microcins and colicins than *fimA*− strains [212]. However, the relative contributions of both pili and bacteriocins to colonization need to be experimentally determined. *E. coli* F-18, a demonstrated colonizer of the mouse large intestine, produces both type I pili and microcin V; among FimA−, FimH− and MccV− mutants, the last was the weakest colonizer of the mouse intestine, indicating a larger effect of MccV in promoting colonization [213].

Pathogens also encode production of bacteriocins, particularly microcins. Clinical isolates that are pathogenic or probably pathogenic, due to an association with a disease state in the patient, frequently produce microcins [34, 37, 39]. Microcins generally, and certain microcins specifically, are found in higher frequencies based on the isolate phylogroup or physical site of isolation in these studies. Among pathogenic isolates, associations between the presence of determinants for microcin production and virulence have been documented in a number of studies [33, 36, 37]. Among faecal *E. coli* isolates from healthy versus diarrhoeic sheep, colicinogeny and microcinogeny (MccV) were more frequently identified in diarrhoea-associated isolates, and MccV production was associated with the ETEC pathotype [199]. However, roughly half of the MccV-producing isolates lacked virulence genes while still inhibiting the enterohaemorrhagic *E. coli* (EHEC) pathogenic strain O157:H7 [199], suggesting thorough analysis of strain characteristics could identify probiotic, microcin-producing strains with good colonization properties but lacking in undesirable virulence characteristics. Horizontal transfer of the plasmid pColV, containing MccV as well as several virulence factors, was recently identified as the probable promoter of clonal expansion of *Salmonella enterica* subsp. *enterica* serovar Kentucky among broiler chickens, and pColV increases both colonization of and virulence (based on a subcutaneous lesion model) towards chickens [27]. A high proportion of genome-sequenced Shiga toxin-producing *E. coli* serogroup O91:H14 isolates from human infections contained microcin-encoding genes in a recent study from Switzerland; isolates from this serogroup also had a higher frequency of virulence genes [214]. Among globally sourced pathogenic *K. pneumoniae* isolates from human liver abscesses, 92 % had a genomic island containing genes encoding MccE492 production as well as various putative virulence determinants [41].

## Microcin-based therapeutics

Microcins have potential as both microbiome therapies and targeted antibacterial drugs, as well as other applications, though they have not been explored as much in these regards as other bacteriocins. Bacteriocins can be applied successfully to regulate microbes and microbial communities. Gram-positive bacteriocins have been utilized routinely as industrial food preservatives since the 1980s [16]. Other varied commercial applications of bacteriocins exist (e.g. a pharmaceutical to prevent pharyngitis; bacterial biopesticides to prevent agricultural crop diseases; teat sanitizer to prevent mastitis in dairy cattle) [215–218]. As bacteriocin research has ramped up in recent decades, with corresponding increases in bacteriocin patent applications, subsequent increases in commercial applications are expected [18]. Biomedical applications comprise the largest portion of published applications research and include applications for infections (systemic, respiratory, oral, stomach, vaginal), cancer, contraception and skin care [18].

Bacteriocins have been historically overlooked for use as targeted therapeutic antibacterials, though this trend is changing. There are several reasons for this. First, pre-antibiotic era research into, for example, use of probiotic, bacteriocin-secreting bacteria to treat gastrointestinal diseases was rapidly put aside with the discovery of small-molecule antibiotics [203, 219]. However, the recent dramatic rise in antibiotic resistance has encouraged a resurgence in explorations of previously abandoned therapies [203, 219]. Second, the advent of the deep-sequencing era, in combination with an explosion in bioinformatic, screening, engineering, and delivery capabilities and greater ecological understanding now enables completely different approaches to solving the problems of novel therapy discovery and applications [203]. Third, improvements in bacterial diagnostics may now enable the targeted use of more narrow-spectrum bacteriocins, whereas previously broad-spectrum antibiotics for unidentified aetiological agents have been preferred [157]. Accordingly, many researchers have promoted a renewed look at bacteriocins, including colicins and microcins, for therapeutic use [157, 158, 220, 221].

Some commercial applications of microcins exist, but most are still in the research stage. Current commercial applications exist in the format of probiotic strains which are confirmed to natively produce microcins (as described in more detail above), though it seems likely that other probiotic and biopesticide strains already in use contain unidentified microcins which may contribute to their colonization capabilities. Much of the research on microcin mechanisms of action, spectra of activity, biochemical properties and ecological roles (described above) can be viewed as necessary foundational work prior to specific applied efforts to regulate individual microbes or communities of microbes.

Though other microcins have been discussed for their potential as therapeutics (see in particular a review on MccB17 [69]), the most significant research progress toward therapeutic application of a microcin, either as a standalone drug or through delivery by an engineered producing strain, is for the Class I microcin, MccJ25. Purified MccJ25 has a favourable safety and stability profile. It is stable in biologically relevant conditions [149, 222], in part due to its lasso conformation, and it maintains this stability in blood without haemolytic activity [151]. Oral delivery of MccJ25 has been tested. Mouse oral administration was shown to reduce ETEC infection and associated symptoms, such as gut inflammation, more effectively than the antibiotic, gentamicin [190]. Importantly, there does appear to be a safety threshold for MccJ25 dosing, above which its beneficial effects in mice are negated, and issues of intestinal imbalance and permeability are observed [189]; this highlights the need for thorough investigations of microcin effects by dosing amount.

Research supports the potential of other applications for MccJ25. It has been suggested as an antibiotic alternative in the case of antibiotics used as feed additives for growth promotion in livestock production. In a direct comparison to colistin provided to weaned pigs, which are highly disease-susceptible, in-feed provision of MccJ25 produced similar or superior results on an array of growth, health and performance metrics [188]. MccJ25 can effectively reduce foodborne pathogens in different food matrices [149], and its derivatives have been suggested for use in food preservation [223].

In a non-antimicrobial microcin application, MccE492 has been examined as a possible cancer treatment due to its ability to kill cancer cells, which is related to its ability to form amyloids [224]. Recently, this effect has been demonstrated *in vivo* for a zebrafish xenograft model, where injected MccE492 was able to shrink tumours formed by human colorectal cancer cells [225]. Bacteriocins as a group are increasingly of interest for their anti-cancer properties, where cytotoxicity of some bacterocions, such as MccE492, is selective towards tumorogenic cells [226].

## Modular and adapted microcins

As the sequence and structural characteristics that govern microcin uptake and activity are further elucidated, it will become easier to modify microcins to suit a specific purpose. This could involve a range of options, which broadly fall into two categories: modular microcins and adapted microcins. Modular microcins (alternatively, hybrid or chimeric microcins) harness their natural modularity to create new microcins or microcin-conjugates using portion(s) of the native microcin. Adapted microcins rely on targeted modification of specific residues or structural features that determine specificity.

**Fig. 4.**
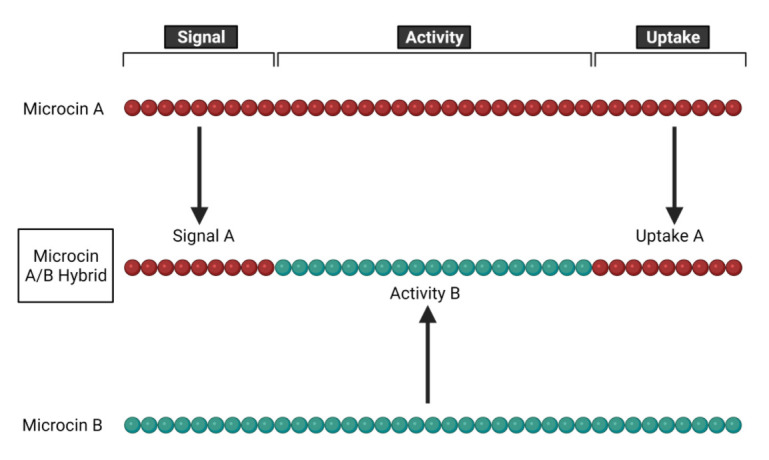
Schematic outlining the creation of a hybrid microcin by combining the activity and uptake domains from two different microcins. Microcin lengths are not to scale. Created with BioRender.com.

The key factor enabling the design of modular and adapted microcins is determining which regions/features control which functionalities. Bacteriocins are commonly modular in structure, where specific domains have been identified which control specific functions. Among colicins, for example, their modular structure, starting from the N-terminus, is: translocation (T) domain, receptor binding (R) domain and cytotoxic (C) domain [110]. Initial research on Class II microcins indicates their design follows similar principles, where the C-terminus determines uptake, while the N-terminus (post-signal cleavage) confers the specific antimicrobial activity (Fig. 4). This was first identified by making MccV and MccH47 chimeras [227]. Activity assays indicated that the last 32 aa of MccV determine uptake by the Cir receptor while the last 11 aa of MccH47, in the presence of additional genetic context for siderophore modification, facilitates addition of the siderophore derivative which determines uptake by either the Cir, FepA or Fiu receptors; the N-terminal remainders determined their activity. Strong similarity between MccV and MccL only in the C-terminal 32 aa was noted [63], and later the receptor for MccL was also confirmed to be Cir [127]. With the identification of the specific domains, it is likely that other microcins could be combined in this modular fashion to swap the naturally occurring modes of uptake and activity (Fig. 4).

There is also evidence suggesting that microcins or portions of microcins can be combined with other bacteriocins or peptides to achieve new functionalities. A modular bacteriocin was created by joining the core peptides (without the signal sequences) of a Class II microcin (MccV) with a Gram-positive Class IIa/YGNGV bacteriocin (enterocin CRL35, Ent35) [228]. Native Ent35 and MccV are active only against Gram-positive and Gram-negative bacteria, respectively. This Ent35–MccV hybrid was active against strains of both Gram-positive and Gram-negative bacteria, including some strains for which neither bacteriocin was active independently.

Microcins have the potential to be combined with non-microcin components as therapeutics. The Gram-positive bacteriocin, nisin, is minimally active towards Gram-negative bacteria. Attaching the 8 aa tail of apidaecin 1b, an AMP produced by honey bees, to nisin increased its membrane permeation, allowing greater access to the lipid II target on the inner membrane and lowering its *E. coli* MIC by two-fold [229]. The current popularity of siderophore and cell-penetrating peptide (CPP) conjugation as tools to enable membrane crossing is similar to the options one could envisage for microcins. Given greater knowledge of microcin diversity and function, their uptake domains could be utilized similarly to siderophores or CPPs, or their activity domains could be conjugated to these other uptake components. Recent FDA approval of cefiderocol, the first siderophore antibiotic [230], bodes well for the acceptance of similar therapeutic concepts based on naturally occurring components. Cefiderocol notably uses conjugation of a siderophore to a member of a long-established class of small-molecule antibiotics (cephalosporins) to enhance its uptake and stability [231].

Regarding the possibility of adapted microcins, this requires detailed knowledge about a microcin beyond identification of specific domains and their functions. Knowledge of the particular residues or structural regions which determine target specificity is needed so they can be altered to fit the needs of the application. Some of this information is becoming available. For example, a three-residue binding region on the first extracellular loop of OmpF was shown to control MccPDI specificity for *E. coli* [232]. Chimeric OmpF composed of N-terminal *E. coli* OmpF and C-terminal *Salmonella enterica* OmpF restored PDI susceptibility to an *E. coli* OmpF deletion mutant [232]. This suggests that the reverse could be done, where key residues on MccPDI are modified to enable it to bind OmpF of *S. enterica *.

Evidence of natural adaptation provides further support for developing adapted microcins. Despite their similarity, homologous microcins MccJ25 and MccY are active against a different range of target strains [116]. Phylogenetic analysis of *Salmonella* species outer membrane receptor FhuA, which both microcins utilize for uptake, produced two clades; one clade corresponds to MccJ25-susceptible isolates and one corresponds to MccY-susceptible isolates [116]. Similarly, phylogenetic analysis to predict target strain susceptibility was performed on the three subunits of the F_0_ sector of ATP synthase [148], the target of MccH47, which is located on the inner membrane and joined to the F_1_ sector located in the cytoplasm. While susceptible *Escherichia *, *Shigella* and *Salmonella* strains grouped together, susceptible *Proteus mirabilis* was phylogenetically more distant than more closely related, but non-susceptible, strains, leading the authors to conclude that uptake may also contribute to susceptibility [148]. In the case of MccC, learning that the terminal residue determined its target specificity for aspartyl-tRNA synthetase led to successful development of new MccC analogues targeting different tRNA synthetases [102]. These results suggest that understanding the factors that naturally determine target specificity can be used to develop microcin-based therapeutics with the specificity of interest.

## Microcin delivery

The selectivity, potency and safety of microcins provides unique opportunities for their use in treating infection and maintaining microbiome health. While purification of sufficient yields required to treat infection remains challenging, the use of bacteria to deliver microcins *in vivo* is showing promise. This can be accomplished either via native secretion by the naturally microcin-producing strain or heterologous secretion by an engineered probiotic.

Native microcin secretion by delivering the producing strain as a probiotic is the only commercially available use of microcin-based therapeutics, as discussed above. However, it does suffer from drawbacks. Microcin production in this case is natively regulated, and the ideal conditions to promote production may be unknown or unachievable. The probiotic strain may carry undesirable features, such as aforementioned associated virulence factors. *E. coli* Nissle, for example, has recently been suggested to have genotoxic and mutagenic activity [233]. Probiotic strains may also be poor colonizers of the site of interest.

Both microcins and double-glycine signal-containing Gram-positive bacteriocins have been secreted heterologously from non-native PCAT/MFP bacteriocin export systems [234–238]. By fusing the core peptide of the desired bacteriocin C-terminally to the native double-glycine secretion signal, heterologous secretion can sometimes be achieved in the bacterial host using its native secretion machinery. Replacement of a native Gram-negative signal sequence with a Gram-positive signal sequence to achieve heterologous Gram-negative peptide bacteriocin secretion in a Gram-positive host, or vice versa, has been demonstrated [234, 235]. The PCAT protease domain is active against diverse substrates, providing key residues of the double-glycine signal are conserved, a key feature which enables this heterologous production [98].

Heterologous secretion by a probiotic *in vivo* provides additional options to engineer targeted and/or regulated delivery. In a unique application to provide targeted microcin delivery, the ‘Seek and Kill’ system was developed in *E. coli* for use against both biofilm and planktonic cells of the human pathogen, *Pseudomonas aeruginosa* [239]. This system incorporates a chemotactic response to a *P. aeruginosa* quorum-sensing signal with secretion of MccS and DNase I via fusion with the endogenous protein YebF. This allows the *E. coli* to deliver MccS, which is not specific for *P. aeruginosa *, in a localized fashion, which may generate a more narrow-spectrum-like effect [239]. Similarly, engineered secretion of microcin H47 from *E. coli *, inducible in response to a biochemical signal of gut inflammation, was used to inhibit *Salmonella* growth *in vitro* [240]. Most recently, engineered production of MccV in conjunction with a quorum-sensing molecule was used to autonomously stabilize a co-culture of the MccV-producer and a susceptible competitor [241]. As the population density of the MccV-producer increased, MccV production decreased in response to the concentration of the quorum-sensing molecule. This allowed the competitor to grow and competitively exclude the MccV-producer, which then ramped up MccV production in response to its own low cell density.

## Future directions

Microcins provide two primary opportunities to advance the development of therapeutics. The first is that, by examining their ecological and mechanistic functions, we can learn how they are natively used to regulate bacterial populations. This can reveal specific pathways for therapeutic exploitation. The second opportunity lies in direct utilization of all or part(s) of a microcin as a therapeutic component or scaffold. Significant recent progress in similar uses of other classes of bacteriocins alongside initial efforts with microcins suggest microcin-based therapeutics are worth exploring. The key limitations to pursuing these opportunities are a lack of known, characterized microcins and difficulties in heterologous production and purification. In particular, systematic searches for novel microcins could propel this field forward. We propose that future work should focus on these issues to advance the field of microcin research.
